# Contrast-Enhanced Ultrasound of Primary Squamous Cell Carcinoma of the Thyroid: A Case Report

**DOI:** 10.3389/fendo.2020.00512

**Published:** 2020-08-11

**Authors:** Sijie Chen, Qinghai Peng, Qi Zhang, Chengcheng Niu

**Affiliations:** ^1^Department of Ultrasound Diagnosis, The Second Xiangya Hospital, Central South University, Changsha, China; ^2^Research Center of Ultrasonography, The Second Xiangya Hospital, Central South University, Changsha, China

**Keywords:** thyroid cancer, thyroid nodules (TNs), thyroid ultrasound (US), primary squamous cell carcinoma, contrast enhanced ultrasound (CEUS)

## Abstract

**Introduction:** Primary squamous cell carcinoma of the thyroid (ThyPSCC) is an extremely rare aggressive malignancy with a poor prognosis. However, almost no report thus far has investigated the microvasculature of ThyPSCC imaged using contrast-enhanced ultrasound.

**Case Report:** A 59-year-old male patient presented to our hospital with progressively worsening hoarse voice symptoms for 20 days and was diagnosed with left unilateral vocal fold palsy. Ultrasonography revealed a solitary marked hypoechoic thyroid nodule with an unclear boundary in the inferior part of the left lobe. Color Doppler flow imaging showed a poor blood flow signal inside this nodule. Contrast-enhanced ultrasound images showed a persistent low peak enhancement of the nodule from its periphery to its center. The time-intensity curve displayed a wash-in time of 10 s, a time to peak of 37 s, a peak signal intensity of 24.5%, and a wash-out time of 70 s for the thyroid tumor. Finally, left hemithyroidectomy of the thyroid tumor was performed, and histopathologic and immunohistochemical evaluations confirmed the diagnosis of ThyPSCC. Postoperatively, the patient received a combination therapy of chemotherapy, radiotherapy, and targeted therapy, but the patient died 4 months after surgery.

**Conclusion:** Primary squamous cell carcinoma of the thyroid is a rare but aggressive malignancy of the thyroid. Herein, we reported a case of ThyPSCC and its ultrasonography and pathologic findings.

## Introduction

Primary squamous cell carcinoma of the thyroid (ThyPSCC) is a rare thyroid malignancy with high aggressiveness and poor prognosis, comprising ~0.1–1% of all primary thyroid carcinomas (1–6). Owing to the rapidly progressing and highly invasive nature of the malignancy, patients with ThyPSCC often present at an advanced stage and are difficult to diagnose in the early stage because of its rare incidence and lack of typical imaging findings (7, 8).

Thyroid ultrasonography and fine-needle aspiration biopsy (FNAB) are the diagnostic tools of choice for evaluating patients with suspected thyroid nodules (9). Contrast-enhanced ultrasound (CEUS), as a relatively novel US technique, is used to investigate the microvasculature of thyroid nodules and improve the diagnostic accuracy of thyroid nodules accompanied by the use of Thyroid Imaging Reporting and Data Systems for ultrasonographic features (10–13). However, very few published studies have reported the use of ultrasonography for ThyPSCC. To our knowledge, this is the first case describing the CEUS findings of ThyPSCC.

## Case Report

**Figure 1:**
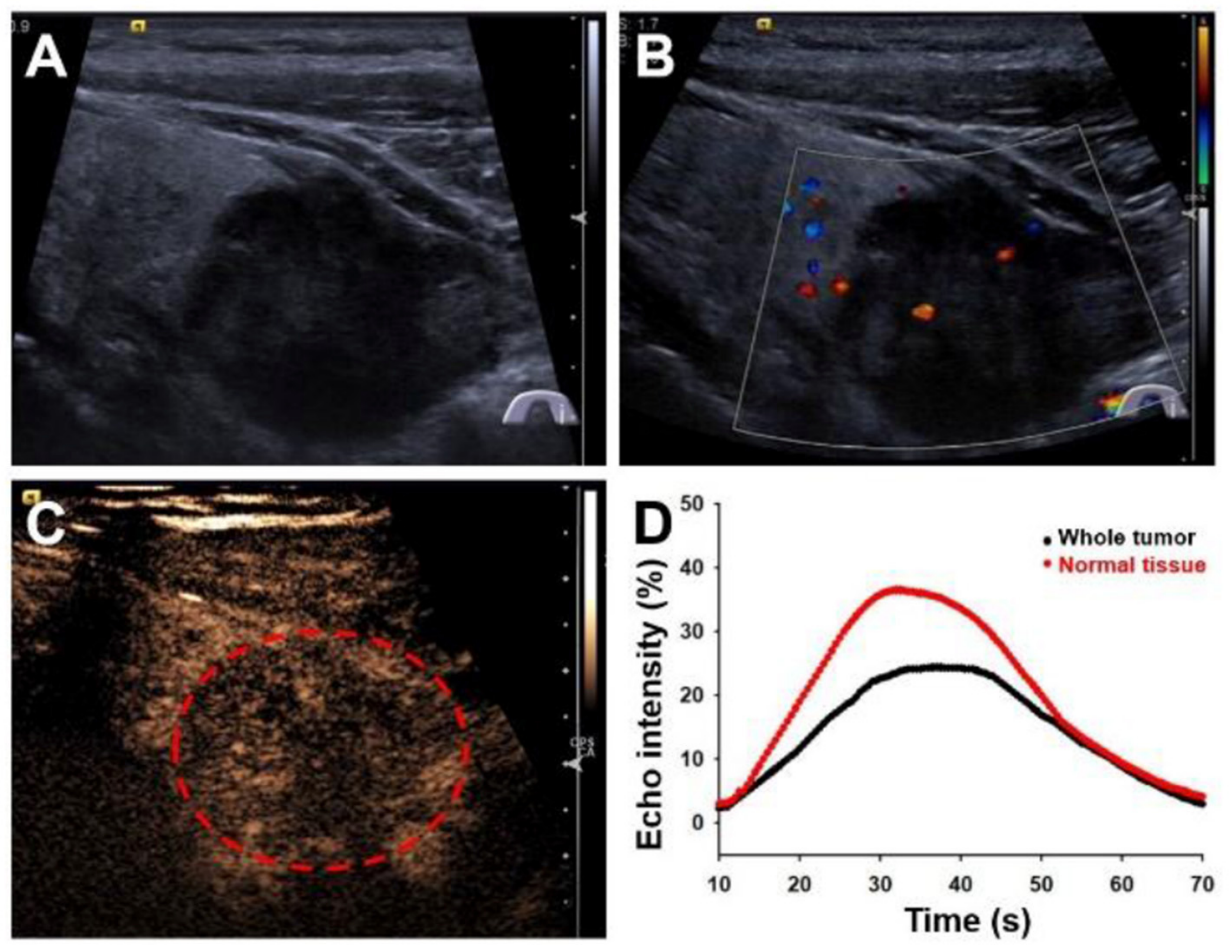
Ultrasonography images of primary squamous cell carcinoma of the thyroid. **(A)** Longitudinal gray-scale sonography revealed a solid marked hypoechoic thyroid nodule in the inferior part of the left lobe. **(B)** Color Doppler flow imaging showed a poor blood flow signal inside this nodule. **(C)** Contrast-enhanced ultrasound image showed a persistent low peak enhancement of the nodule at 37 s. **(D)** Time-intensity curves displayed the wash-in time of 10 s, TTP of 37 s, peak signal intensity of 24.5%, and wash-out time of 70 s for the thyroid tumor.

A 59-year-old male patient presented to our hospital with progressively worsening hoarse voice symptoms for 20 days and was diagnosed with left unilateral vocal fold palsy. A high-resolution ultrasound instrument (Siemens Acuson S3000, Mountain View, CA, USA) equipped with a 4- to 9-MHz linear probe was used. Thyroid ultrasonography revealed a solitary 3.1 × 2.8 × 2.6-cm^3^ marked hypoechoic thyroid nodule with an unclear boundary in the inferior part of the left lobe (Figure 1A). This nodule exhibited many malignant ultrasound features, such as solid components, hypoechogenicity, and microlobulated margins. Color Doppler flow imaging (CDFI) showed poor blood flow signals in the nodule (Figure 1B). Contrast-enhanced ultrasound was performed with a bolus intravenous injection of 3.0 mL of SonoVue (Bracco, Milan, Italy) followed by 5 mL of saline. Contrast pulse sequencing technology was used, and the time-intensity curves (TICs) of the nodule were calculated. The nodule began to be slowly enhanced from the periphery to the center at 10 s (wash-in time), and the enhancement reached its peak [time to peak (TTP)] at 37 s with a peak intensity of 24.5%. Then, the nodule slowly declined until all the microbubbles washed out at 70 s (Figures 1C,D). Based on its malignant conventional ultrasound features and the poor microvasculature revealed by CEUS, we inferred that the nodule was a malignant tumor.

**Figure 2:**
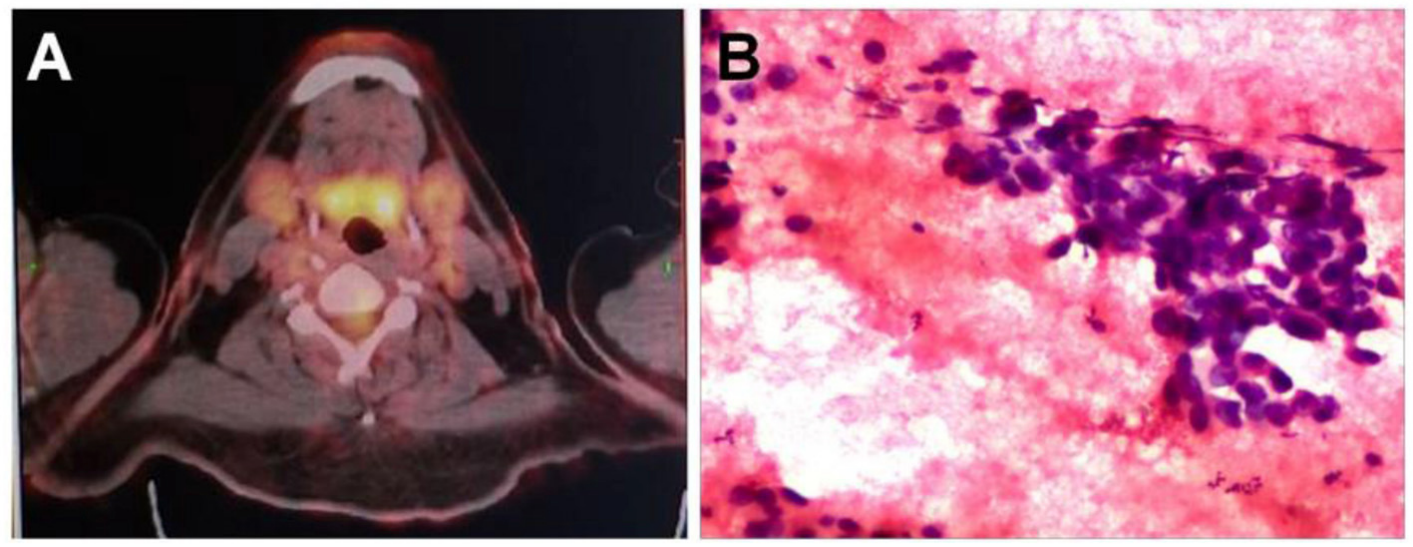
**(A)** A positron emission tomography–computed tomography scan showed increased ^18^F-fluorodeoxyglucose metabolism in the left neck mass. **(B)** Preoperative fine-needle aspiration cytology of the mass demonstrated a few sheets of malignant-looking tumor cells with giant deep stained nuclei (hematoxylin and eosin, magnification × 400).

**Figure 3:**
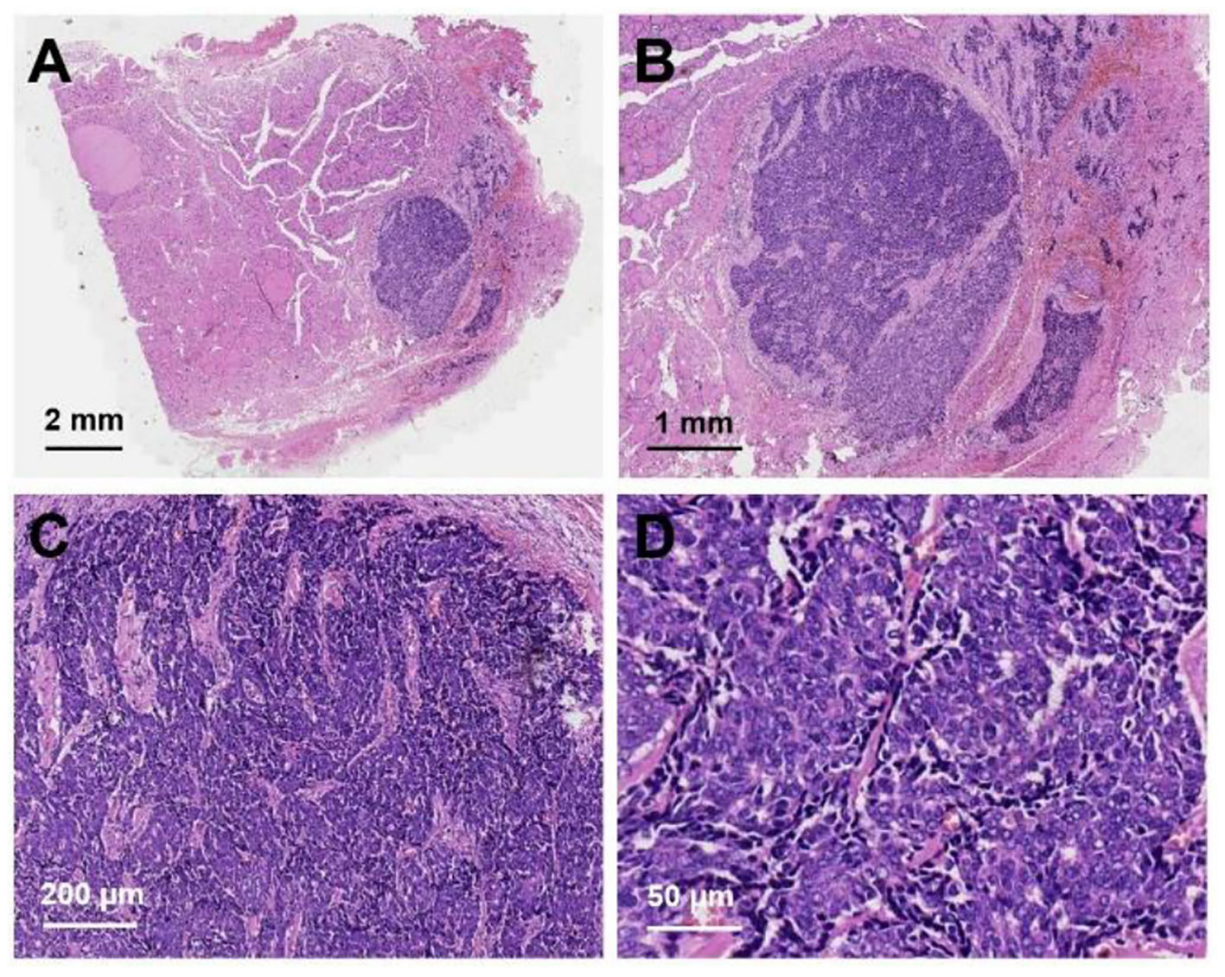
Hematoxylin and eosin staining of primary squamous cell carcinoma of the thyroid: **(A)** magnification × 8, **(B)** magnification × 20, **(C)** magnification × 100, **(D)** magnification × 400.

**Figure 4:**
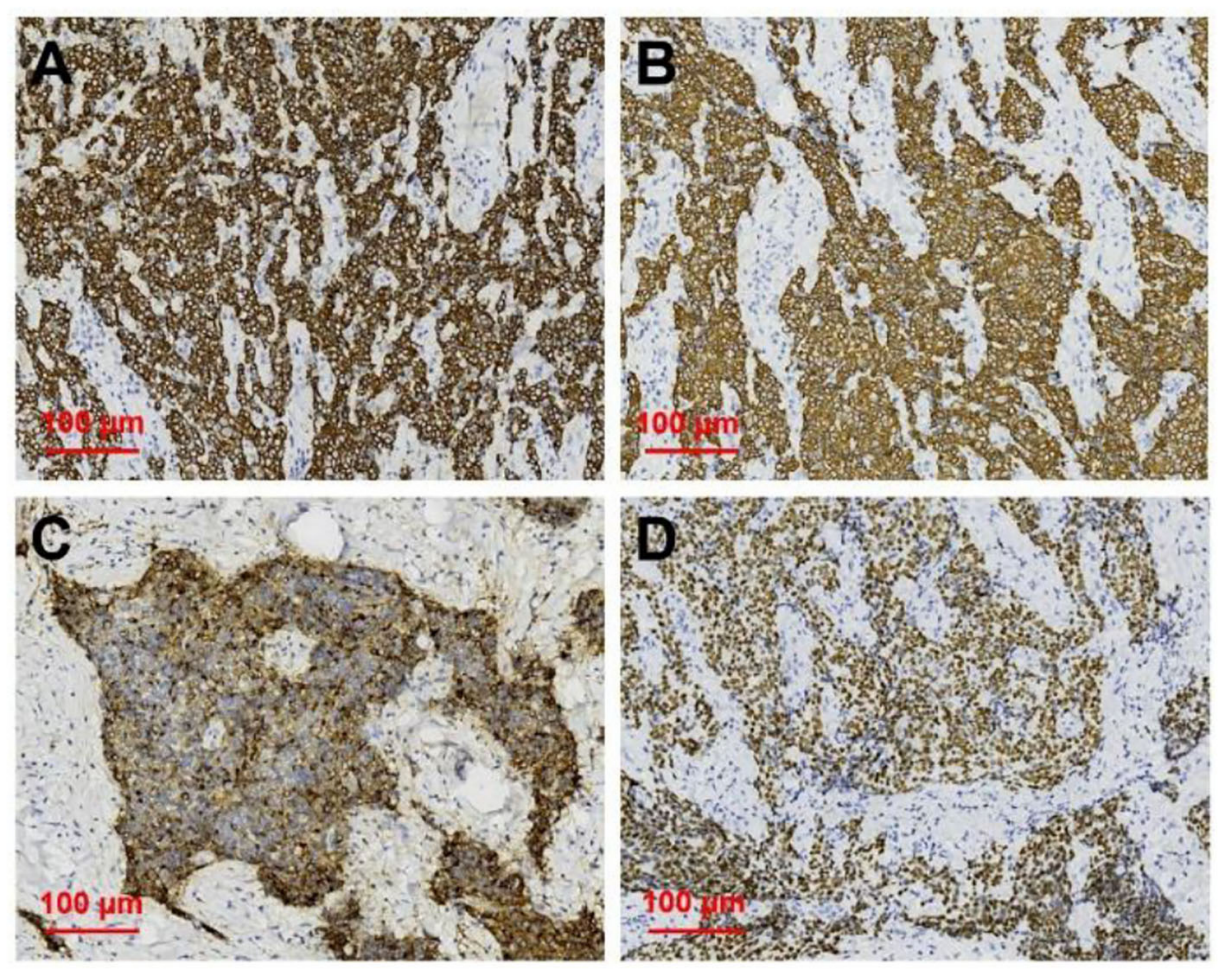
Immunohistochemical staining of primary squamous cell carcinoma of the thyroid (magnification × 200). Immunohistochemical staining for **(A)** CK19, **(B)** CK5/6, **(C)** EMA, **(D)** p40, all of which were deeply stained (positive).

**Figure 5:**
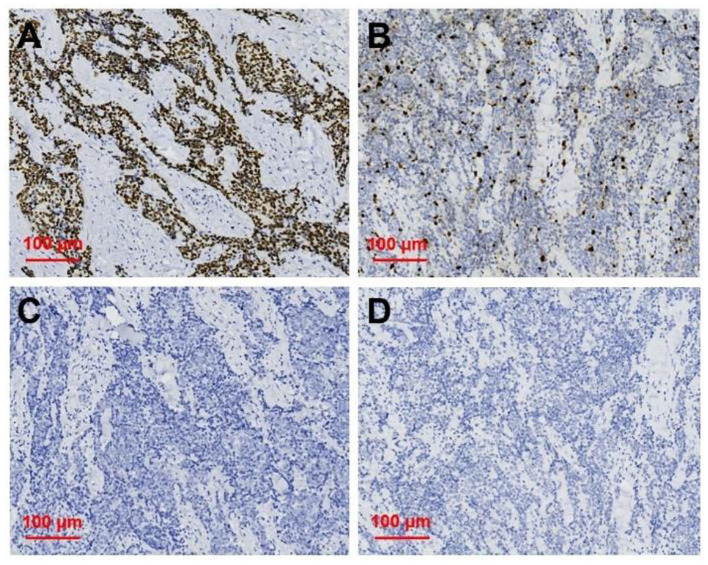
Immunohistochemical staining of primary squamous cell carcinoma of the thyroid (magnification × 200). Immunohistochemical staining for **(A)** p63, **(B)** Ki 67, **(C)** TG, **(D)** TTF-1, and p63 was deeply stain (positive); Ki67 proliferation index was 30%; TG and TTF-1 did not stain (negative).

After neck ultrasonography, the positron emission tomography–computed tomography was carried for evaluating the situation of distant metastases. Positron emission tomography–computed tomography showed a mass with increased glucose metabolism in the inferior part of the left thyroid lobe (Figure 2A), which indicated it as a malignant mass, whereas there was no evidence of lymph nodes metastasis and distant metastases. Then, ultrasonography-guided FNAB was performed for the left thyroid mass immediately. Cytologic examination by fine-needle aspiration (FNA) revealed sheets of tumor cells with giant deep-stained nuclei (Bethesda category V) (Figure 2B). Finally, a left hemithyroidectomy of the thyroid tumor was undertaken. The lower edge of the tumor reached the upper mediastinum, and the depth of the tumor invaded the esophagus and trachea, which could not be completely removed. According to the eighth edition of the American Joint Committee on Cancer/Tumor Lymph Node Metastasis (TNM) staging system (14), the patient was in TNM stage III (T4a N0 M0). Histopathological examination of hematoxylin and eosin staining showed that a carcinoma in the inferior part of the thyroid lobe (Figure 3A) had no obvious palisade arrangement, intercellular bridges, or keratinization with a cancer pearl (Figures 3B–D). Immunohistochemically, tumor cells were positive for cytokeratin 19 (CK19, Figure 4A), cytokeratin 5 and 6 (CK5/6, Figure 4B), epithelial membrane antigen (EMA, Figure 4C), p40 (Figure 4D), p63 (Figure 5A), and Ki-67 (30%+, Figure 5B) and negative for thyroglobulin (TG, Figure 5C) and thyroid transcription factor 1 (TTF-1, Figure 5D). In view of these findings, the tumor was diagnosed as poorly differentiated ThyPSCC. Postoperatively, the patient received two cycles of chemotherapy with docetaxel/cisplatin, intensity-modulated radiotherapy, and nimotuzumab-targeted therapy. However, the patient died 4 months after surgery.

## Discussion

Primary squamous cell carcinoma of the thyroid is a thyroid malignancy with extremely rare incidence, and the clinical diagnosis and treatment guidelines for this disease have no consensus (4). The biological behavior of ThyPSCC is aggressive, and the prognosis is poor, with a median overall survival of 4–24 months, which depends on the different tumor grades (1). Yang et al. using the Surveillance, Epidemiology, and End Results Program database, reported that poorly differentiated tumor grade occupied the highest percentages of all graded tumors, and the median survival was 4 months, which is similar to the survival time in our case (1).

High-frequency ultrasound, as the basic imaging modality in the diagnosis of thyroid nodules, has found gradually increasing differentiated thyroid cancers over recent years (15, 16). The ultrasonography imaging findings of ThyPSCC have seldom been published. Regarding the ultrasonography findings, Chen et al. (17) reported that ThyPSCC presented as a thyroid mass with eggshell calcification, peripheral soft tissue with a blurred margin, and minimal vascular signals on CDFI sonography. In the case of Jang et al. (7), ThyPSCC presented as a large, well-defined, lobulated, heterogeneously hypoechoic mass with diffuse microcalcifications on ultrasonography. Kondo et al. (18) reported that a well-differentiated ThyPSCC showed a cystic hypoechoic mass with a smooth margin and rapidly grew with margin change blurring in 1 year. In our case, this poorly differentiated ThyPSCC presented as a solitary marked hypoechoic thyroid mass with an irregular margin and unclear boundary with a normal thyroid. The irregular margin and unclear boundary with normal thyroid corresponded to tumor invasion with adjacent tissue infiltration, which is consistent with the findings during the operation that tumor invasion with the esophagus cannot be completely removed. Poor blood flow signals on CDFI sonography and persistent hypoenhancement on CEUS of the mass are consistent with squamous cell carcinoma, which has no obvious vascularity on pathologic examination.

Many studies have investigated the application of CEUS to improve the diagnostic accuracy of thyroid nodules, despite its usage in ThyPSCC being scarce. Zhang et al. (15) found that high/circular/equal enhancement indicated benign thyroid nodules, and low enhancement indicated malignant thyroid nodules. Ma et al. (19) investigated whether incomplete, no ring or heterogeneous enhancement, later wash-in time, and low peak intensity on CEUS were independent risk factors in predicting malignant thyroid nodules. Deng et al. (20) detected that papillary thyroid carcinomas (PTCs) exhibited low enhancement, a lower peak signal intensity, and a lower area under the curve (AUC) than peripheral thyroid parenchyma on CEUS (13, 20). In our study, the TICs of CEUS for ThyPSCC showed a wash-in time of 10 s, a TTP of 37 s, a peak signal intensity as low as 24.5%, and a wash-out time of 70 s. This is similar to the results of PTCs with a slow wash-in time, a lower peak signal intensity, and a lower AUC, as in previous reports (13). To our knowledge, no reports on CEUS imaging findings of ThyPSCC have appeared in the English-language literature. According to Jang et al. (7), ThyPSCC showed a large heterogeneously enhancing thyroid mass with a large central non-enhancing portion on enhanced CT, which corresponded well with the squamous cell carcinoma portion with a necrotic portion in pathologic staining. Because of the rapid growth of squamous tumor cells, relatively few interstitial blood vessels in tumors were related to the low peak signal intensity and low AUC on CEUS.

With increasing malignancy in squamous cell carcinoma, the typical squamous cell carcinoma findings of intercellular bridges and keratinized cancer pearl can decrease or disappear. Immunohistochemical staining is useful in diagnosing primary thyroid cancer. In this case, positivity for CK5/6 and EMA and negativity for TTF-1 and TG expression predicted squamous cell carcinoma derivation and excluded the possibility of these common tumors (3, 21). Further positivity for p63 and Ki67 expression as poor prognostic markers was associated with its poorly differentiated tumor grade (7, 22).

## Conclusion

Primary squamous cell carcinoma of the thyroid is an extremely rare tumor, and very few studies describe its ultrasonographic imaging findings. It is difficult to establish a clinical guideline for diagnosis. Our case presents the CEUS features of ThyPSCC, indicating that the TICs of ThyPSCC are similar to the enhancing parameters of PTCs with a slow wash-in time, a lower peak signal intensity, and a lower AUC.

## Data Availability Statement

The datasets generated for this study are available on request to the corresponding author.

## Ethics Statement

The studies involving human participants were reviewed and approved by the Ethics Committee of Second Xiangya Hospital, Central South University, China. The patients/participants provided their written informed consent to participate in this study. Written informed consent was obtained from the individual(s) for the publication of any potentially identifiable images or data included in this article.

## Author Contributions

All authors listed have made a substantial, direct and intellectual contribution to the work, and approved it for publication.

## Conflict of Interest

The authors declare that the research was conducted in the absence of any commercial or financial relationships that could be construed as a potential conflict of interest.
